# Initiated Chemical Vapor Deposition Kinetics of Poly(4-aminostyrene)

**DOI:** 10.3389/fbioe.2021.670541

**Published:** 2021-04-16

**Authors:** Alexandra Khlyustova, Rong Yang

**Affiliations:** Robert Frederick Smith School of Chemical and Biomolecular Engineering, Cornell University, Ithaca, NY, United States

**Keywords:** polymer, kinetics, deposition rate, initiated Chemical Vapor Deposition, activation energy, thin films, 4-aminostyrene, controlled drug release

## Abstract

*Initiated* Chemical Vapor Deposition (iCVD) is a free-radical polymerization technique used to synthesize functional polymer thin films. In the context of drug delivery, the conformality of iCVD coatings and the variety of functional chemical moieties make them excellent materials for encapsulating pharmaceutics. Poly(4-aminostyrene) (PAS) belongs to a class of functionalizable materials, whose primary amine allows decoration of the delivery vehicles with biomolecules that enable targeted delivery or biocompatibility. Understanding kinetics of PAS polymerization in iCVD is crucial for such deployments because drug release kinetics in thin-film encapsulation have been shown to be determined by the film thickness. Nevertheless, the effects of deposition conditions on PAS growth kinetics have not been studied systematically. To bridge that knowledge gap, we report the kinetics of iCVD polymerization as a function of fractional saturation pressure of the monomer (i.e., P_m_/P_sat_) in a dual-regime fashion, with quadratic dependence under low P_m_/P_sat_ and linear dependence under high P_m_/P_sat_. We uncovered the critical P_m_/P_sat_ value of 0.2, around which the transition also occurs for many other iCVD monomers. Because existing theoretical models for the iCVD process cannot fully explain the dual-regime polymerization kinetics, we drew inspiration from solution-phase polymerization and proposed updated termination mechanisms that account for the transition between two regimes. The reported model builds upon existing iCVD theories and allows the synthesis of PAS thin films with precisely controlled growth rates, which has the potential to accelerate the deployment of iCVD PAS as a novel biomaterial in controlled and targeted drug delivery with designed pharmacokinetics.

## Introduction

Polymers have been widely used as the encapsulation material to enable controlled release of pharmaceutics due to their desirable properties such as biocompatibility, mechanical flexibility, rich functional moieties, and cost-effective manufacturing (Khlyustova et al., 2020). Although polymer particles and hydrogels have remained the most prevalent drug delivery vehicles (Perrotta et al., 2018), polymer thin films have emerged in recent years as an alternative that could enable programmable drug release kinetics through precise film thickness control (Ghasemi-Mobarakeh et al., 2019; Sayin et al., 2019; Decandia et al., 2020).

While existing polymer thin films in drug delivery have commonly relied on solution synthesis and processing (Ré, 2006), solvent-free techniques such as initiated Chemical Vapor Deposition (iCVD) offer distinct advantages over the conventional approaches, including elimination of solvents and improved control over film thickness. Dichloromethane, ethyl acetate, and silicone oil have been used in the spray drying of poly(D,L-lactic acid) to obtain a thin film that encapsulated bovine serum albumin for a parenteral delivery system (Gander et al., 1996). To avoid acutely toxic solvents such as dichloromethane, water and ethanol have been used in encapsulation of albendazole (used for treatment of echinococcosis). Nevertheless, this approach is limited to water-soluble polymers such as polyvinyl alcohol, polyvinyl pyrrolidone, and hydroxypropyl methylcellulose (Alanazi et al., 2007), which commonly lead to high dissolution rates and thereby high and hard-to-control release rates *in vivo* (Ni et al., 2017). In addition to solvent toxicity, the choice of appropriate solvents is further constraint by potential solvent-mediated degradation of active therapeutic ingredients (Christian et al., 2016; Qiu et al., 2020). Furthermore, the solution methods allow limited control over thickness of the encapsulating polymer films. To address the limitations of the solution-based techniques, the solvent-free polymerization technique, iCVD, has been employed to synthesize encapsulation coatings in drug delivery (Coclite et al., 2013; Sayin et al., 2019). To date, the versatile iCVD technique has been used to encapsulate highly soluble antibiotic gentamicin (Decandia et al., 2020), to enable the controlled release of phenytoin and indomethacin (Christian et al., 2018; Werzer et al., 2019), and to fabricate wound dressing that releases clotrimazole (Ghasemi-Mobarakeh et al., 2019). Copolymers of methacrylic acid (MAA) has enabled enteric drug delivery via its pH-dependent swelling, which increased the mesh size in alkaline environments (Lau and Gleason, 2007). Using a similar iCVD polymer, camptothecin was released at a slow rate at pH of 1.8 and a controllable high rate at pH of 7.4 (McInnes et al., 2012). In the aforementioned study of gentamicin encapsulation, copolymers of MAA retained the antibacterial activity (Decandia et al., 2020).

Initiated Chemical Vapor Deposition enables all-dry free radical polymerization. During iCVD, the substrates to be coated (e.g., films of pharmaceutics) are placed on a temperature-controlled stage and thus kept under room-temperature and solvent-free conditions throughout the one-step polymerization and film formation, retaining the full activities of labile pharmaceutics. The iCVD precursors (i.e., initiator and monomers) enter the reactor chamber under medium vacuum, where the monomers adsorb onto the substrate surface (e.g., a drug film) and the initiator breaks down into radicals (Lau and Gleason, 2006b; Zhao et al., 2017), which initiate polymerization upon surface impingement (Tenhaeff and Gleason, 2008; Decandia et al., 2020). Furthermore, iCVD is often implemented with an *in situ* interferometry that allows nanometer-scale control of the thickness of the film grown on a reflective substrate (e.g., a silicon wafer). Despite that film thickness control on reflective substrates, polymer growth kinetics on non-reflective substrates (such as porous substrates or drug films) could differ considerably from that on a silicon wafer (Cheng et al., 2020). As a result, deep understanding of the iCVD polymerization kinetics is required to enable precise control of the thickness of encapsulation layers and of drug release kinetics.

Here, we demonstrated improved understanding of the iCVD kinetics using the monomer 4-aminostyrene (AS). AS was chosen because its primary amine group enables further functionalization by biomolecules including antibodies, enzymes and genetic materials (e.g., RNAs and DNAs). For example, amide bond between a primary amine and a carboxylic acid has been commonly used in biocoupling reactions such as peptides (Li et al., 2020) or antibody immobilization (Maraldo and Mutharasan, 2007). Another example is a reaction between primary amine and aldehyde via nucleophilic addition, which enables functionalization by chitosan and other monosaccharides (Butler et al., 2003). Furthermore, poly(4-aminostyrene) (PAS) is known to be pH-responsive, hydrophobic in neutral pH and soluble in acidic pH (Wang et al., 2014), enabling accelerated drug release under acidic environments such as tumor tissues with pH as low as 5.7 (Cao et al., 2019).

Despite the plethora of potential applications in drug delivery and biomaterials, the growth kinetics of iCVD PAS remain elusive. Details about the iCVD polymerization of AS, such as the rate law with respect to monomer concentration and stage temperature, have not been reported, potentially limiting the ability to achieve controlled drug release kinetics. To address that challenge, this report provides detailed understanding of the effects of the fractional saturation pressure of monomer (P_m_/P_sat_) and stage temperature on the rate of PAS film growth. Distinct from the previously observed iCVD kinetics (Lau and Gleason, 2006a, b), where rate of polymerization depends on P_m_/P_sat_ in a quadratic fashion, we captured two regimes of the rate dependence. In a narrow range of low P_m_/P_sat_ (specifically, P_m_/P_sat_ < 0.2), the rate of deposition was proportional to (P_m_/P_sat_)^2^, which has been observed in solution free-radical polymerization when primary radical termination dominates (Bamford et al., 1959) (taking the approximation that P_m_/P_sat_ is comparable to [M], bulk monomer concentration). At high P_m_/P_sat_, rate of deposition depends linearly on (P_m_/P_sat_), consistent with the solution free-radical polymerization when disproportionation or combination is the dominating termination mechanism. That has also been corroborated by several previous reports on iCVD polymerization (Tenhaeff and Gleason, 2008; Ozaydin-Ince and Gleason, 2009), but the systematic characterization of the full continuum from quadratic to linear transition has not been achieved. In addition, we uncovered the critical P_m_/P_sat_ value of 0.2, around which the quadratic-to-linear transition occurs for many iCVD monomers (Gupta and Gleason, 2006; Ozaydin-Ince and Gleason, 2009). The reported kinetics study enables the accurate prediction and detailed tuning of the growth kinetics of a highly functionalizable polymer PAS synthesized using a state-of-the-art all-dry polymerization technique, iCVD. The capability to predict and control the growth of PAS thin films has the potential to enable novel designs of targeted and smart drug delivery vehicles with well understood release kinetics and thereby pharmacokinetics, accelerating materials design, and deployment in healthcare.

## Materials and Methods

### Chemicals and Materials

4-aminostyrene (97%, Sigma-Aldrich), tert-butyl peroxide (TBPO, 98%, Sigma-Aldrich) were both used as received without further purification.

### The iCVD Setup

**FIGURE 1:**
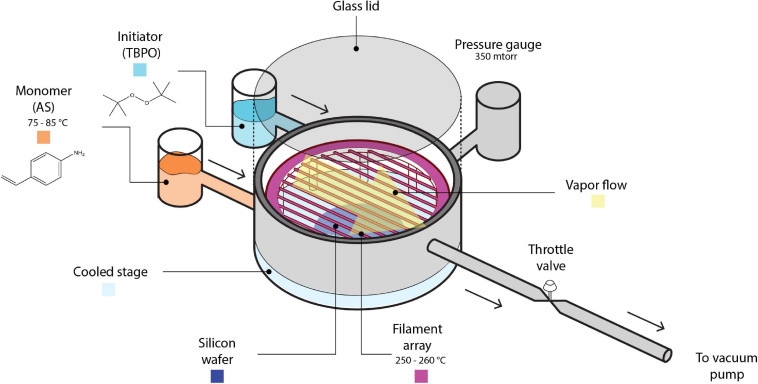
The schematic of initiated Chemical Vapor Deposition reactor.

All polymerization reactions were done in a custom-built iCVD reactor chamber (335 mm diameter, 51 mm height, schematic shown in Figure 1), which was evacuated by an E2M40 rotary vane vacuum pump (Edwards Vacuum, United Kingdom). During deposition, AS was heated to 75–85°C and total reactor pressure was kept at 0.350 Torr, well within the common pressure range used in iCVD processes (i.e., 0.1–1 Torr) (Tenhaeff and Gleason, 2008) while being on the low end of that range. The pressure was selected in this work in consideration of potential applications for the polymer thin films of PAS in drug delivery, where conformal coatings (which are commonly associated with lower P_m_/P_sat_ and thus lower total pressure) are most desirable. Furthermore, the pressure chosen was shown to avoid condensation on the reactor lid and stage, which tend to happen at higher pressure values even with the low monomer flow rate used in this work. The filament temperature was kept at 250–260°C for all experiments, selected to be consistent with previous reports on PAS (Xu and Gleason, 2010). P_m_/P_sat_ was changed by varying stage temperature or monomer flow rate (see Supplementary Material section “Process Conditions for Initiated Chemical Vapor Depositions of Poly(4-aminostyrene)” for details). The initiator flow rate was kept constant at 0.50 ± 0.02 sccm. Due to the non-volatile nature of AS, inert gas was not included such that measurable deposition rates can be obtained. The variation in total flow rate caused by the absence of inert gas is ≤0.25 sccm among all depositions, which is small enough to not change the deposition kinetics significantly, as evidenced by the excellent fit between experimental data and theoretical predictions that were made based on this assumption (see Figure 3).

**FIGURE 2:**
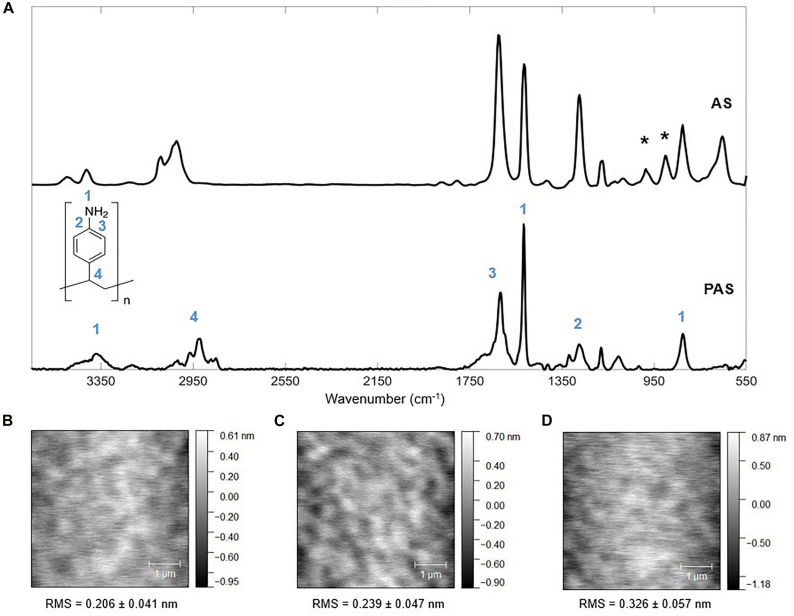
Chemical and topographical characterization of PAS. **(A)** Fourier-transform infrared spectroscopy (FTIR) of AS standard (National Institute of Standards and Technology) and PAS thin film deposited via iCVD. Asterisks (*) in the AS spectrum correspond to vinyl bonds. The thickness of the FTIR sample is 133.67 ± 4.36 nm deposited using P_m_/P_sat_ of 0.324. Atomic force microscopy (AFM) images of a 5 × 5 μm view of iCVD PAS thin films deposited at P_m_/P_sat_ of **(B)** 0.132, **(C)** 0.163, and **(D)** 0.235, with the root-mean-square (RMS) roughness labeled on each plot. Data = Mean ± SD, *n* = 2. The scale bar represents 1 μm.

**FIGURE 3:**
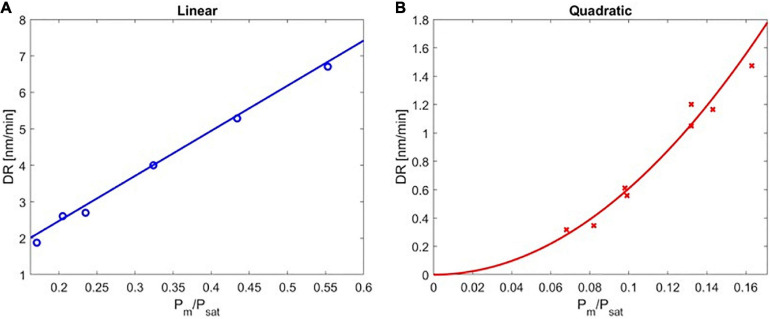
The deposition rate of poly(4-aminostyrene) as a function of P_m_/P_sat_ in the **(A)** linear, and **(B)** quadratic regimes.

### Chemical Characterization

Fourier-transform infrared spectroscopy (FTIR) was used to determine film composition. Thickness of the thin films was measured by ellipsometry. See Supplementary Material section “Chemical and Topographical Characterization” for details.

## Results and Discussion

### Chemical Composition and Topography of the iCVD PAS Thin Films

To confirm the obtainment of the PAS molecular structure via iCVD polymerization, FTIR was applied to iCVD-deposited PAS with the thickness of ∼ 200 nm. The spectra of iCVD PAS and an AS standard (from National Institute of Standards and Technology) are shown in Figure 2A along with the chemical structure of PAS. FTIR spectra were collected for samples deposited under each set of conditions listed in Supplementary Table 1, and they were identical to Figure 2A. Therefore, a representative spectrum, collected on samples deposited with the P_m_/P_sat_ value of 0.324 is shown here. In the spectrum of PAS, the absence of peaks corresponding to =CH and =CH_2_ bending (which occurred at around 900 cm^–1^ and labeled with asterisks for AS) proved that polymerization was complete. Furthermore, despite the observed difference in peak intensities between the AS monomer standard and iCVD PAS (e.g., the relative peak intensities of 1 and 3 in Figure 2A), the spectrum of iCVD PAS agreed well with the literature (Cha et al., 2016). Each peak that corresponds to a functional moiety in the PAS was labeled with a numerical index to match the labeling on the molecular structure in Figure 2A. For example, there were three peaks corresponding to the N-H bond, all labeled with “1.” N-H stretching led to a few weak peaks located at ∼ 3400 cm^–1^. At around 1516 cm^–1^ there was a medium peak due to N-H bending of the primary amine. The peak at 825 cm^–1^ corresponded to the N-H out-of-plane bending (i.e., N-H wagging). The peak located at 1271 cm^–1^ (labeled with “2”) corresponded to the C-N stretching adjacent to the phenyl group. The peak at 1620 cm^–1^ (labeled with “3”) was due to C=C stretching in the aromatic ring. The peak at around 3000 cm^–1^ (labeled with “4”) corresponded to C-H stretching that was a result of the combined effect of the C-H next to the phenyl group and those in the polymer backbone. Therefore, the FTIR spectrum supported that PAS was obtained successfully by using iCVD.

Atomic force microscopy (AFM) in Figures 2B–D captures PAS topography at different P_m_/P_sat._ The surface roughness of all films was <0.5 nm, confirming the ultra-smoothness of the iCVD polymer films. The surface roughness increased slightly from 0.206 ± 0.041 nm for P_m_/P_sat_ of 0.132 to 0.326 ± 0.057 nm for P_m_/P_sat_ of 0.235. That mild increase in surface roughness upon increasing P_m_/P_sat_ can be explained by a minor increase in the sticking probability of free radicals, i.e., a higher likelihood of surface impingement upon collision of radicals on the monomer-adsorbed surface, hence, greater rate of localized initiation of polymerization, which ultimately led to slightly increased surface roughness. The same effect reduces conformality of thin films deposited over nano-structured substrates, as shown in Supplementary Material section “Conformality of PAS in 3D Structures.”

### Rate Laws for the iCVD of PAS

Similar to solution-based free radical polymerization, the kinetics iCVD have been shown to be controlled by the surface concentration of monomers, [M] (moles/m^3^), which is proportional to the monomer fractional saturation pressure, P_m_/P_sat_, i.e., the ratio of monomer partial pressure to its saturation pressure at the temperature of the stage (Lau and Gleason, 2006a, b). P_m_/P_sat_ is proportional to the monomer surface concentration because the surface adsorption of monomers in iCVD has been shown to follow the Brunauer–Emmett–Teller (BET) isotherm (Lau and Gleason, 2006a, b). The BET theory describes the equilibrium of multilayer physisorption, where each surface site (i.e., the surface area occupied by a single molecule) is assumed to have the same adsorption energy and only molecules in adjacent layers are assumed to have interactions (Brunauer et al., 1938). Applying the BET theory to describe the monomer physisorption in iCVD yields (Lau and Gleason, 2006a, b):

(1) $$\left[\text{M}\right]=\frac{\rho_{M}}{M}\times \frac{c\times \frac{P_{m}}{P_{s\,a\,t}}}{\left(1-\frac{P_{m}}{P_{s\,a\,t}}\right)\times \left(1-\left(1-c\right)\times \frac{P_{m}}{P_{s\,a\,t}}\right)}$$

(2) $$\lim_{\frac{P_{m}}{P_{s\,a\,t}}\to 0}\left[M\right]\approx \frac{\rho_{M}}{M}\times c\times \frac{P_{m}}{P_{s\,a\,t}}$$

where ρ_*M*_ is density of liquid monomer, which is 1017 kg/m^3^ here (i.e., the density of AS) (Sharma et al., 2019); *M* is the molar mass of AS, i.e., 0.119 kg/mol; c is the BET constant, which represents the energy of monolayer adsorption (Ambroz et al., 2018). By assuming the enthalpy of desorption for the first layer (i.e., at the monomer-substrate interface) to be constant and greater than that of the second layer (i.e., the heat of vaporization), the BET constant can be calculated using the Arrhenius law for desorption (Duncan, 1949):

(3) $$c=\exp\left[\frac{\Delta \,H_{\text{des}}-\Delta \,H_{\text{vap}}}{R\,T_{\text{stage}}}\right]$$

where *T*_stage_ is stage temperature in iCVD,*R* is a gas constant {i.e., 8.314 [J/ (mol K)]}, △*H*_*des*_ and △*H*_*vap*_ are enthalpy of desorption and heat of vaporization, respectively. The heat of vaporization of AS has been calculated from simulations to be 47.6 ± 3.0 kJ/mol (ChemSpider, 2020); whereas the reported enthalpy of desorption for styrene, which is 61 kJ/mol, was used to approximate △*H*_*des*_ for the monomer AS (Ranke and Weiss, 2000). As such, the BET constant, *c*, for AS was calculated to be 1.005.

Consistent with the existing theories of iCVD (Lau and Gleason, 2006a, b), here we assumed that the rate of deposition is independent of the initiator concentration. That assumption has been recently validated in experiments, where radicals were shown to be generated in such abundance that the chemisorption of radicals, i.e., the initiation step of the iCVD polymerization, was not the rate-limiting step under regular iCVD conditions (Şimşek and Karaman, 2020). As a result, the rate of iCVD polymerization can be expressed in terms of P_m_/P_sat_, as follows:

(4) $$R_{\text{poly}}\,\left(\text{flat}\right)=k\times {\left[M\right]}^{\alpha }=k^{\prime }\times {\left[\frac{P_{m}}{P_{\text{sat}}}\right]}^{\alpha }$$

where α is the order of reaction; α = 1 for termination via disproportionation or combination, and α = 2 for termination by primary radical termination, consistent with the reported rate laws for solution-based free radical polymerization (Bamford et al., 1959). Previously, the rate law for iCVD kinetics has been expressed as either a quadratic dependence (Lau and Gleason, 2006b, a) or a linear dependence (Janakiraman et al., 2015); whereas here, we adopted this unifying expression to capture the full continuum from quadratic to linear transition as discussed below. The reaction rate coefficient, *k*, can be calculated using the Arrhenius relation (Laidler, 1984):

(5) $$k=A\times \text{exp}\,\left(-\frac{E_{a,\mathrm{overall}}}{R\,T_{\text{stage}}}\right)$$

where A is the pre-exponential factor, depicting the collision frequency between reacting molecules; *R*, *T*_*stage*_, and *k* are as described previously. The Eqs 4 and 5 combined offered an approach to correlate the rate of polymerization (experimentally measured via deposition rate, see Supplementary Material section “Calculation of Rate of Polymerization” for the detailed derivation for calculating rate of polymerization using deposition rate) with the monomer surface concentration (experimentally measured via P_m_/P_sat_), and thereby to regress for the rate constant (*k*) and order of reaction (α):

(6) $$\left[\text{M}\right]=\frac{\rho_{M}}{M}\times \frac{c\times \frac{P_{m}}{P_{s\,a\,t}}}{\left(1-\frac{P_{m}}{P_{s\,a\,t}}\right)\times \left(1-\left(1-c\right)\times \frac{P_{m}}{P_{s\,a\,t}}\right)}$$

where *k*′ is an empirical rate constant, which took on the values of 11.517 and 68.08 in Eqs 7 and 8, respectively.

#### The Order of Reaction for the iCVD of PAS

The experimentally obtained deposition kinetics of PAS are depicted in Figure 3. The rate of deposition was plotted as a function of P_m_/P_sat_ and divided into two regimes: the linear regime under high P_m_/P_sat_ (Figure 3A) and the quadratic regime under low P_m_/P_sat_ (Figure 3B). The transition point between the linear and the quadratic regimes was determined using a statistical approach (see Supplementary Material section “Quantification of the Transition Point” for details).

DR of PAS increased linearly with P_m_/P_sat_ from 0.171 to 0.553, indicating the rate of the polymerization had first-order dependence on the monomer surface concentration (the maximum value of P_m_/P_sat_ that could be obtained without causing local condensation in the iCVD reactor was 0.553). It is important to note that DR of PAS was extremely slow (<7 nm/min), consistent with the previously reported rate of deposition (Gleason and Xu, 2013). This first-order kinetic dependence agreed with solution-based free-radical polymerization, where the polymerization rate is proportional to the concentration of monomer [M] when chain termination is dominated by disproportionation or combination. That linear dependence on [M] originated from the pseudo-steady state approximation (Hiemenz and Lodge, 2007), where the rate of monomer consumption is assumed to represent the rate of chain growth, and the rates of initiation and termination are assumed to be equal. In addition, the rate of initiation is assumed to be dominated by primary radical dissociation, which is monomer-independent, while initiation of polymer chain can be neglected due to its fast reaction rate (long chain approximation). In this linear regime, the data in Figure 3A can be regressed with a high coefficient of determination (*R*^2^ = 0.955, with the maximum error in this region being ±0.209 nm/min for P_m_/P_sat_ of 0.171, corresponding to an 8% range) by the following equation:

(7) $$\text{DR}=11.517\,\left(\frac{P_{m}}{P_{\text{sat}}}\right).$$

Consistent with previous iCVD studies, DR increased with greater P_m_/P_sat_ values (Lau and Gleason, 2006a). The empirical rate constant for the linear regime, i.e., 11.517 nm/min, was comparable to the reported values for other iCVD polymerizations. For example, poly(vinylpyrrolidone), also a non-volatile monomer, had an empirical rate constant of approximately 5.26 nm/min in the linear regime (Janakiraman et al., 2015), while a more reactive monomer, cyclohexyl methacrylate, led to an empirical rate constant of ∼100 in the linear regime (Baxamusa and Gleason, 2008). Comparatively, AS belongs to a class of monomers with relatively slow deposition kinetics among the iCVD monomers reported to date.

DR of PAS followed a quadratic dependence on the P_m_/P_sat_ in the range of 0.082 to 0.171. The resulting DR was even slower than that in the linear regime (<2 nm/min in the quadratic regime), which was expected due to the low concentration of surface monomers. This second-order kinetic dependence also agreed with solution-based free-radical polymerization, when chain termination is dominated by primary radical termination (Bamford et al., 1959). Under these conditions, the pseudo-steady state approximation applies and thus rate of initiation is equal to rate of primary radical termination. As a result, the concentration of growing chains is proportional to the concentration of monomers, leading to the quadratic dependence of polymerization rate on monomer concentration (Goodner and Bowman, 1999). In this quadratic regime, the data in Figure 3B can be regressed as follows (*R*^2^ = 0.994, with the maximum error of ±0.153 nm/min, occurred at P_m_/P_sat_ of 0.163 ± 0.008).

(8) $$\text{DR}=69.08\,{\left(\frac{P_{m}}{P_{\text{sat}}}\right)}^{2}$$

The second-order dependence of DR for low P_m_/P_sat_ values was also consistent with the previous studies, where the deposition of ethyl acrylate was found to vary with P_m_/P_sat_ in a quadratic fashion when P_m_/P_sat_ < 0.04 (Lau and Gleason, 2006a, b).

Note that the range of P_m_/P_sat_ values shown in Figure 3B were obtained using a combination of two strategies, i.e., varying P_m_ and/or varying *T*_stage_ (due to the limited flow rate range one could achieve for a non-volatile monomer like AS). The separate datasets for each strategy (i.e., varying P_m_/P_sat_ or substrate temperature) were included in Supplementary Figure 2. The error bar in Supplementary Figure 2D represents the results of four independent depositions performed under the same conditions, i.e., the highest P_m_/P_sat_ (0.163 ± 0.008) in the quadratic regime with the DR of 1.473 ± 0.113 nm/min. Despite the difference in experimental conditions, the datasets followed an identical quadratic trend with a high coefficient of determination, further confirming the validity of the assumptions.

#### The Rate Constants for the iCVD of PAS

To provide reliable predictions for the iCVD rate of polymerization or DR in both regimes, Eq. 6 was used to regress for values of the rate constant, k, in both regimes. In this section, only datasets obtained under identical stage temperatures were used (see Supplementary Table 1 samples PAS3 and PAS10–PAS14), with the consideration that the rate constant, k, is a function of temperature (Eq. 5). The iCVD reaction constant was calculated to be 0.064 ± 0.006 L/ (mol s) for quadratic regime and 0.111 s^–1^ for the linear regime. The difference in those constants can be attributed to different termination mechanisms in these regimes. The constant in quadratic regime is twofold smaller since primary radical termination proceeds faster as primary radicals scavenge the growing chain, thus stopping the polymerization reaction leading to slower growth rate.

In solution phase, this reaction rate constant, k, in quadratic regime represents the overall effects of the propagation constant (k_*p*_) multiplied by chain initiation constant (k_*i*_) over primary radical termination constant (k_tp_), while in linear regime, the reaction rate constant takes into account the propagation constant (k_*p*_), initiator dissociation constant (k_*d*_) and its efficiency (f), as well as termination constant for combination and disproportionation (k_*t*_) (Odian, 2004). In addition, the propagation constant is a function of the type of initiator, solvent, and the reaction temperature, making a direct comparison to iCVD polymerization challenging, especially given that monomer surface adsorption instead of radical initiation is often the rate-limiting step in iCVD, unlike the case for solution polymerization.

### The Activation Energy for the iCVD of PAS

**FIGURE 4:**
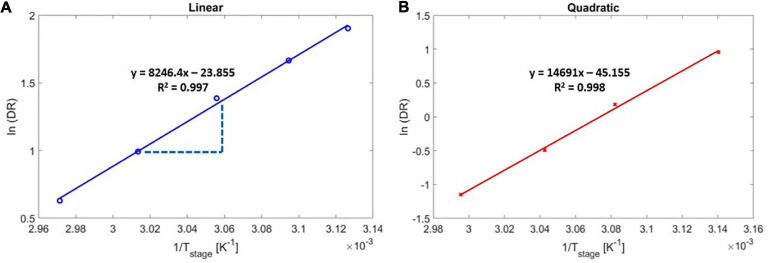
Arrhenius deposition rate as a function of substrate temperature for poly(4-aminostyrene) activation energy calculation in **(A)** linear, and **(B)** quadratic regimes.

The experimental activation energy was determined by measuring the DR under different stage temperatures (Figure 4), and by regressing for the overall activation energy, *E*_*a,overall*_, using Eq. 5. Figure 4 shows the Arrhenius plot for the DR as a function of 1/*T*_stage_ for the linear (Figure 4A) and the quadratic (Figure 4B) regimes. As reported in the literature (Lau and Gleason, 2006a), the DR of PAS decreased with increasing stage temperature in both regimes, indicating negative values for the activation energy, which is a result of the adsorption-limited deposition kinetics (monomer adsorption is exothermic). According to the Arrhenius correlation (Eq. 5), the slopes in Figure 4 represented -*E*_*a*,*overall*_/R, and the overall activation energy for the iCVD of PAS was thus determined to be −68.56 kJ/mol for the linear regime and −122.15 kJ/mol for the quadratic regime.

Those experimentally obtained values of the iCVD activation energy were validated by an estimate made using the following correlation (Lau and Gleason, 2006a):

(9) $$E_{a,\text{overall}}^{\prime }=E_{a,\text{prop}}-\alpha \,\Delta \,H_{\text{des}}$$

where $E_{a,o\,v\,e\,r\,a\,l\,l}^{\prime }$ is the estimated overall activation energy; *E*_*a,prop*_ is the activation energy of the free radical polymerization of AS, which was assumed to be zero due to the adsorption-limited kinetics of iCVD (Lau and Gleason, 2006a); △*H*_*des*_ is the aforementioned heat of desorption; α is the order of reaction.

Using Eq. 9 with the order of reaction, α = 1 (indicating the linear regime) yields a predicted value of △*H*_*des*_ of 61 kJ/mol. There is the small difference between the calculated activation energy, −61 kJ/mol, and experimentally measured activation energy, −68.56 kJ/mol, likely occurred due to collection of experimental data near the P_m_/P_sat_ transition point of 0.163 ± 0.008.

In the quadratic regime (i.e., α = 2), the experimental activation energy (*E*_*a*,*overall*_ = −122.15 kJ/mol) represented a strikingly close match to the estimated value of $E_{a,o\,v\,e\,r\,a\,l\,l}^{\prime }=$122 kJ/mol, where $E_{a,o\,v\,e\,r\,a\,l\,l}^{\prime }$ was calculated using Eq. 9 and the previously reported △*H*_*des*_ value of 61 kJ/mol (Ranke and Weiss, 2000). That close match validated the accuracy of the experimental measurements and confirmed that the activation energy associated with free radical polymerization can be neglected.

This overall activation energy was also the smallest reported to date for iCVD due to the high enthalpy of desorption of the monomer AS. Despite that strong tendency to surface-adsorb, the DR of PAS was slow, which was likely a result of the small pre-exponential factor, A. Using Eq. 5, the pre-exponential factor, A, was determined to be 2.291 × 10^10^ s^–1^ for the linear regime (calculated using data in Figure 4A). This value fell on the low-end of surface-controlled reactions, which typically have a pre-exponential factor A > 10^9^ s^–1^ (Genieva et al., 2010). The small pre-exponential factor and deposition kinetics of PAS can be explained by the partial transfer of electrons from the primary amine group to the vinyl group, reducing the probability of free radical initiation (Goikhman et al., 2011).

Interestingly, the critical P_m_/P_sat_ value of 0.2, around which the transition from quadratic to linear regime occurred for the iCVD of PAS, could apply to other iCVD chemistries. For example, P_m_/P_sat_ of 0.24 for poly(1H,1H,2H,2H-perfluorodecyl acrylate) (Gupta and Gleason, 2006) (PPFDA) and P_m_/P_sat_ of 0.20 for poly(ethylene glycol diacrylate) (Ozaydin-Ince and Gleason, 2009). The generality was likely a result of the transition from a monolayer or less of adsorbed monomers, and thus primary radical termination dominates, to multilayer monomer adsorption, which favors disproportionation or combination termination mechanisms.

## Conclusion

The kinetics of the iCVD polymerization of AS were studied in great details. With low surface monomer concentration, the rate of polymerization/deposition varied with the surface monomer concentration in a quadratic fashion. Upon increasing the fractional saturation pressure of the monomer, i.e., P_m_/P_sat_, to values above 0.163 ± 0.008, the rate of polymerization/deposition depends linearly on the surface monomer concentration. The existence of these two regimes is consistent with the solution-based free radical polymerization, where the linear rate dependence applies to polymerization reactions terminated via disproportionation or combination and the quadratic rate dependence applies to those terminated by primary radical termination. Those distinct kinetic regimes were further corroborated by measurements of the overall iCVD activation energies in each regime, where the value of 68.56 kJ/mol was obtained for the linear regime and that of 122.15 kJ/mol for the quadratic regime. The latter represented a striking match with the theory-derived estimation (122 kJ/mol) made by using the enthalpy of adsorption for polystyrene. To enable facile estimation of the DR under varying deposition conditions, the iCVD rate constants in each regime were calculated using the experimental data reported here. The overall rate constant for the linear regime was 0.111 s^–1^ and that for the quadratic regime was 0.064 L/ (mol s). The constants were different due to dominance of different termination mechanism in each regime. However, it is still not clear how iCVD constants correlate with solution-phase constants since iCVD is assumed to be initiator-independent. Future studies are needed to figure out contribution of initiation, propagation, and termination constants in the expression of iCVD reaction constants. These rate constants could be used to estimate deposition rates using the corresponding formulation of the rate law for each regime. The two distinct termination mechanisms that led to the quadratic and linear kinetics likely had significant impact on the molecular weight and polydispersity of the iCVD PAS. Nevertheless, despite our best effort, PAS cannot be deposited in sufficient quantities for a molecular-weight analysis using conventional approaches like gel permeation chromatography (GPC). This challenge is also evident from the literature as such molecular weight data do not exist for iCVD-synthesized PAS, despite the long history and high utility of this polymer. Quantifying the molecular weight and polydispersity of iCVD polymers synthesized using monomers with low volatility or reactivity is a key issue that require novel approaches, which will be the focus of our future studies.

The unifying kinetic model and kinetic parameters reported here will enable the precise estimation of the polymer thin film growth kinetics based on conditions or the *a priori* selection of synthesis conditions that lead to the desirable rate of polymerization. The precise control of polymerization kinetics and facile selection of synthesis conditions thus could allow the accelerated design and deployment of PAS-based drug delivery vehicles with controlled and/or predictable pharmacokinetics.

## Data Availability Statement

The original contributions presented in the study are included in the article/Supplementary Material, further inquiries can be directed to the corresponding author.

## Author Contributions

AK conducted the iCVD depositions and all characterization experiments. AK and RY contributed to the experimental design, data analysis, and results interpretation, and drafted the manuscript and/or edited it critically for important intellectual content. Both authors contributed to the article and approved the submitted version.

## Conflict of Interest

The authors declare that the research was conducted in the absence of any commercial or financial relationships that could be construed as a potential conflict of interest.
