# Supplementary material for: Initiated Chemical Vapor Deposition Kinetics of Poly(4-aminostyrene)
Source: Front Bioeng Biotechnol. 2021 Apr 16;9:670541. doi: 10.3389/fbioe.2021.670541 (PMC8085358; doi:10.3389/fbioe.2021.670541)
Supplement: Supplementary file 1 [file Data_Sheet_1.pdf]

*Supplementary Material*

# Initiated Chemical Vapor Deposition Kinetics of Poly(4-aminostyrene)

**Alexandra Khlyustova[1], Rong Yang[1*]**

[1] Robert Frederick Smith School of Chemical and Biomolecular Engineering, Cornell University, Ithaca, New York 14853, United States of America

*** Correspondence:**
Rong Yang
E-mail: ryang@cornell.edu

**Keywords:** *polymer, kinetics, deposition rate, initiated Chemical Vapor Deposition, activation energy, thin films, 4-aminostyrene, controlled drug release.*

## 1 Process Conditions for initiated Chemical Vapor Depositions of Poly(4-aminostyrene)

Table S1 represents the summary of poly(4-aminostyrene) (PAS) initiated Chemical Vapor Deposition (iCVD) deposition conditions to figure out activation energy and dependence on $P_m/P_{sat}$ in linear and quadratic regimes (Substrate Temperature Series 1 and 2).

**Table S1.** iCVD process conditions for PAS. Substrate Temperature Series 1 corresponds to Figure 4A, and Substrate Temperature Series II corresponds to Figure 4B.

**Substrate Temperature Series 1**

| Sample | Total Pressure (Torr) | Monomer Flow Rate (sccm) | Initiator Flow Rate (sccm) | Total Flow Rate (sccm) | $T_{stage}$ (°C) |
|---|---|---|---|---|---|
| PAS1 | 0.35 | 0.30 | 0.52 | 0.82 | 50.0 ± 0.6 |
| PAS2 | 0.35 | 0.30 | 0.52 | 0.82 | 58.7 ± 0.5 |
| PAS3 | 0.35 | 0.30 | 0.52 | 0.82 | 54.1 ± 0.6 |
| PAS4 | 0.35 | 0.30 | 0.52 | 0.82 | 63.4 ± 0.8 |
| PAS5 | 0.35 | 0.30 | 0.52 | 0.82 | 46.7 ± 0.7 |

**Substrate Temperature Series 2**

| Sample | Total Pressure (Torr) | Monomer Flow Rate (sccm) | Initiator Flow Rate (sccm) | Total Flow Rate (sccm) | $T_{stage}$ (°C) |
|---|---|---|---|---|---|
| PAS6 | 0.35 | 0.07 | 0.50 | 0.57 | 45.3 ± 0.8 |
| PAS7 | 0.35 | 0.07 | 0.51 | 0.58 | 51.3 ± 1.1 |
| PAS8 | 0.35 | 0.07 | 0.51 | 0.58 | 55.5 ± 1.2 |
| PAS9 | 0.35 | 0.07 | 0.51 | 0.58 | 60.7 ± 0.8 |

**Monomer Series**

| Sample | Total Pressure (Torr) | Monomer Flow Rate (sccm) | Initiator Flow Rate (sccm) | Total Flow Rate (sccm) | $T_{stage}$ (°C) |
|---|---|---|---|---|---|
| PAS10 | 0.35 | 0.09 | 0.50 | 0.59 | 54.0 ± 1.0 |
| PAS11 | 0.35 | 0.10 | 0.50 | 0.60 | 54.0 ± 1.0 |
| PAS12 | 0.35 | 0.12 | 0.50 | 0.62 | 54.0 ± 1.0 |
| PAS13 | 0.35 | 0.06 | 0.50 | 0.56 | 54.0 ± 1.0 |
| PAS14 | 0.35 | 0.05 | 0.50 | 0.55 | 54.0± 1.0 |

PAS thin films were deposited on 100 mm diameter silicon wafers (Pure wafer, USA). The pressure in the iCVD reactor chamber was controlled by a throttle butterfly valve (MKS Instruments, USA), and measured by a manometer (Baratron, MKS Instruments, USA). On the top of the chamber, a glass lid was located to allow for *in situ* laser interferometry (He-Ne laser, JDSU, USA), visual observation and placement of substrates onto the cooled stage. The reactor chamber also consisted of a filament array (B&K Precision, USA), which heated nickel-chromium wires (80% Ni/20% Cr) that were located 28 mm above the stage. The array was used to decompose initiator into free radicals, which then further reacted with AS monomer. The stage temperature was controlled by an Accel 500 LC chiller (Thermo Fisher, USA). Both stage and filament temperatures were monitored by using type K thermocouple (Omega Engineering, USA). During deposition, AS was heated at 75-85 ºC, and it was flown into the reactor chamber through a heated line maintained at 105 ºC. The desired flow rate of AS was set by using a needle valve (Swagelok, USA). The initiator, TBPO, was kept at a room temperature, and its flow rate was controlled by using a mass flow controller (MKS Instruments, USA). To determine $P_m/P_{sat}$ of PAS, the room temperature of 25 °C and boiling point of 213.5 °C, and standard pressure of 760 torr with vapor pressure of 0.0415 torr were used (Xu and Gleason, 2010).

Three sets of data on deposition kinetics of PAS were collected to figure out the dependence of DR on $P_m/P_{sat}$ at different regimes. To figure out activation energy in linear regime, AS flow rate was set to 0.30 sccm, initiator flow rate was 0.52 sccm, while stage temperature was varied from 46.7 to 63.4 °C. The [I]/[M] ratio was kept constant at 1.72 ± 0.02. For the 2$^{nd}$ series in quadratic regime, AS flow rate was set to 0.07 sccm, initiator flow rate was 0.50 ± 0.01 sccm, while the stage temperature was changed from 45.3 to 60.7 °C. The [I]/[M] ratio was kept constant at 7.18 ± 0.13.

For the 3$^{rd}$ set the deposition rate (DR) was studied at low-monomer environment, where the quadratic dependence on monomer concentration is expected. For this experiment, the stage temperature was set to 54.0 ± 1.0 °C, initiator flow rate was 0.50 sccm, while the flow rate of AS was changed from 0.05 to 0.12 sccm, thus the total flow rate was 0.55 ± 0.07 sccm, respectively.

## 2 Chemical and Topographical Characterization

Fourier-transform infrared spectroscopy (FTIR) (Bruker Vertex V80V Vacuum FTIR system with cooled MCT and room temperature DTGS detectors) was used to determine final composition of coated substrates. The spectra were acquired over 400 - 4000 $cm^{-1}$ with the resolution of 4 $cm^{-1}$ and 256 total scans. The spectra were analyzed, and the baseline was corrected by using OPUS software (Bruker). Surface roughness was measured by using an Asylum-MFP3D-BIO Atomic Force Microscope (AFM). Scans were recorded across 5 x 5 µm regions at 0.5 Hz in AC air tapping mode. The thickness of the thin films on a flat surface (silicon wafers) was measured by variable angle spectroscopic ellipsometry (Alpha-SE, J.A. Woollam) at three different angles (65º, 70º and 75º) with a wavelength range from 315 to 718 nm. To determine the deposition rate (DR), the coating thickness of PAS, which was measured by ellipsometry, was divided by the total deposition time.

## 3 Conformality of PAS in 3D Structures

To evaluate conformality of PAS coating in 3D structures, iCVD was used to coat Anodic Aluminum Oxide Membranes (AAO) membranes at different $P_m/P_{sat}$ values. AAO membrane was chosen as a substrate because of its nanoscale pore sizes (averaging around 100 nm here), which is a desirable length scale in drug delivery applications (Mitchell et al., 2020). Figure S1A depicts a pristine AAO membrane with average pore size of 107.81 ± 12.02 nm and pore length is 4.5 μm. Figure S1B shows the AAO membrane after PAS deposition performed at a low $P_m/P_{sat}$ value of 0.064. The resultant coating thickness inside pores is 16.32 nm judging from the SEM image of the top surface of coated membranes. Furthermore, to demonstrate coating conformality at a distance from the top surface, we used ion-milling to remove 250 nm of materials (membranes and coatings) and examined the membrane samples with SEM again. Figure S1C shows that at 250 nm beneath the membrane top surface, the coating thickness is 11.14 nm, comparable to the top surface, thus conforming conformality of PAS coating at low $P_m/P_{sat}$. For the AAO membrane coated at a higher $P_m/P_{sat}$ value of 0.221, the resultant coating thickness was 12.25 nm on top surface of the membrane (Figure S1D) and 5 nm after ion-milling away the top 250 nm of materials (Figure S1E). This observation of reduced conformality upon increasing $P_m/P_{sat}$ value is consistent with the previous iCVD reports (Baxamusa and Gleason, 2008). Hence, it is desirable to coat PAS at $P_m/P_{sat}$ values below 0.1 to get the most conformal coating for drug delivery applications.

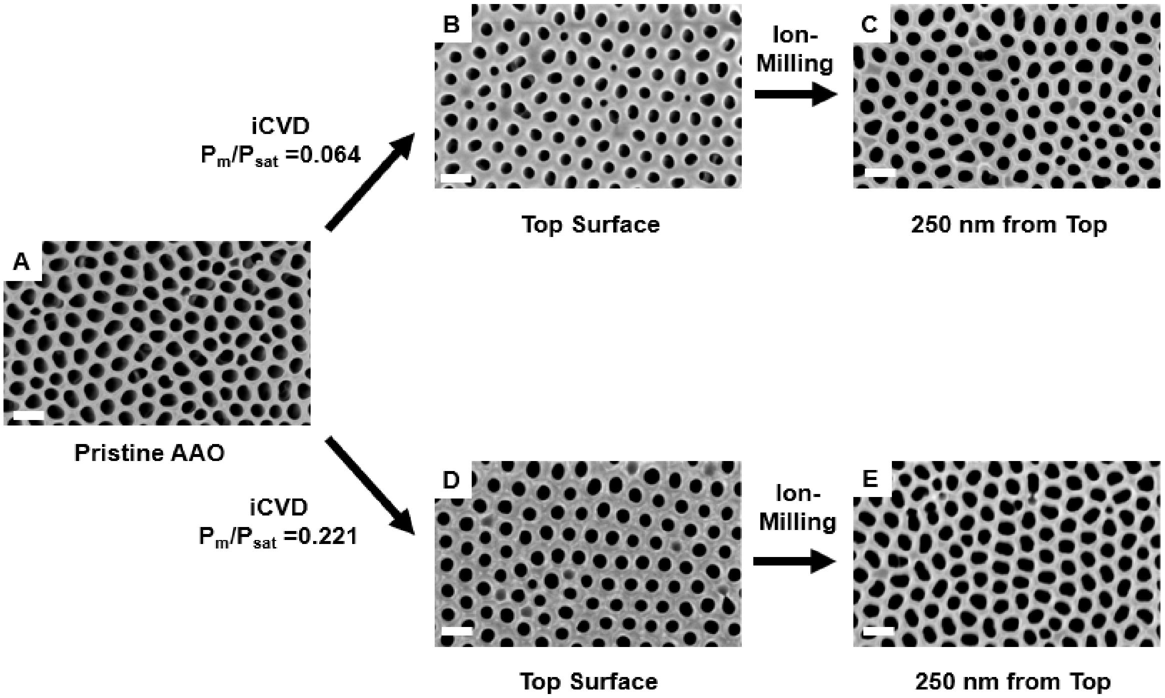


**Figure S1.** Evaluation of conformality of PAS coating in AAO Membranes: (A) Scanning Electron Microscopy (SEM) image of pristine AAO membrane; SEM image of PAS-coated AAO membrane (B) top surface and (C) after 250 nm ion-milling at $P_m/P_{sat}$ of 0.064; SEM image of PAS-coated AAO membrane (D) top surface and (E) after 250 nm ion-milling at $P_m/P_{sat}$ of 0.221. The scale bar represents 200 nm.

## 4 Calculation of Rate of Polymerization

The rate of polymerization was calculated from the experimental data to regress to iCVD reaction constant, k (see equation 6 in the main report). The rate of polymerization was calculated from DR using the equation below:

$$R_{poly}(flat) = \frac{\rho_p DR}{h_{ml} M} \qquad [1]$$

where DR is deposition rate (= thickness/deposition time), $\rho_p$ is polymer density [approximated to be the same as poly(styrene) density of 1050 kg/m$^3$] (Spencer and Gilmore, 1949), and $h_{ml}$ is monolayer thickness. $h_{ml}$ has been previously determined experimentally to be $1.46*10^{-9}$ m for ethyl acrylate (Lau and Gleason, 2006a, 2006b), while its molecular length is known to be $1.70*10^{-9}$ m calculated by ChemBioDraw Chem 3D suite (Asatekin and Gleason, 2011). Respectively, the molecular size of AS is $2.21*10^{-9}$ m, so $h_{ml}$ for AS can be approximated to be $1.90*10^{-9}$ m assuming that the ratio of $h_{ml}$(ethyl acrylate) over molecular length is the same as this ratio for AS.

## 5 Quantification of the Transition Point

As the dependence of DR on $P_m/P_{sat}$ transitioned from quadratic to linear, the experimental data was compared to the regressed linear trend using the following criterion:

$$\left|\frac{DR_{linear} - DR_{exp}}{DR_{exp}}\right| < \left|\frac{DR_{max} - DR_{avg}}{DR_{avg}}\right|_{max} \qquad [2]$$

where $DR_{linear}$ is calculated using equation 7 in terms of $P_m/P_{sat}$; $DR_{exp}$ is the deposition rate measured experimentally at said $P_m/P_{sat}$; $DR_{avg}$ is the average deposition rate for 4 replicates, and $DR_{max}$ is the upper bound of the experimentally recorded deposition rate (occurred at $P_m/P_{sat}$ = 0.163), which is expected to be within or near the linear regime. The right-hand side represents the maximum possible variance in an individual measurement of DR, which remains within the tolerance to be considered part of the linear regime. Therefore, when the difference between $DR_{predict}$ (i.e., the DR calculated using equation 7 by assuming linear regime applies) and $DR_{exp}$, the measured DR, and normalized by $DR_{exp}$, exceeds the tolerance defined above, the $DR_{exp}$would be considered to fall outside the linear regime. The value of right-hand side was a constant, which is equal to 9.7 %.

The points with $P_m/P_{sat}$ smaller than 0.171 did not follow the linear trend. Specifically, the point at $P_m/P_{sat}$ of 0.163 was the last point in the quadratic regime from the linear analysis. At $P_m/P_{sat}$ of 0.163 $DR_{predict}$ value was 1.877 nm/min, while $DR_{exp}$ was 1.473 nm/min, which led to discrepancy of 27.4 %. Therefore, for PAS the transition point between the quadratic and linear regime is expected to occur around $P_m/P_{sat}$ of 0.163 ± 0.008, which corresponds to [I]/[M] ratio of 4.344 in the vapor phase, while in solution-phase free radical polymerization, this transition point was experimentally shown to occur [I]/[M] > 1% (Lebreton and Boutevin, 2000). Such a large difference in transition point between iCVD and solution-phase free radical polymerization, might be due to small fraction of activated during iCVD depositions. iCVD initiation is known to be limited by the temperature of filament, which restricts the amount of nucleation sites formed on the surface (Mao and Gleason, 2004).

**Figure S2.** (A) Deposition rate of PAS as a function of $P_m/P_{sat}$ for three sets of experiments, where (B) the stage temperature was changed in linear regime, and (C) in quadratic regime; (D) the monomer flow rate was changed in quadratic regime, while other parameters were kept constant. The error bar represents the results of 4 independent depositions performed under the same conditions, i.e., the highest $P_m/P_{sat}$ (0.163 ± 0.008) in the quadratic regime with the DR of 1.473 ± 0.113 nm/min.

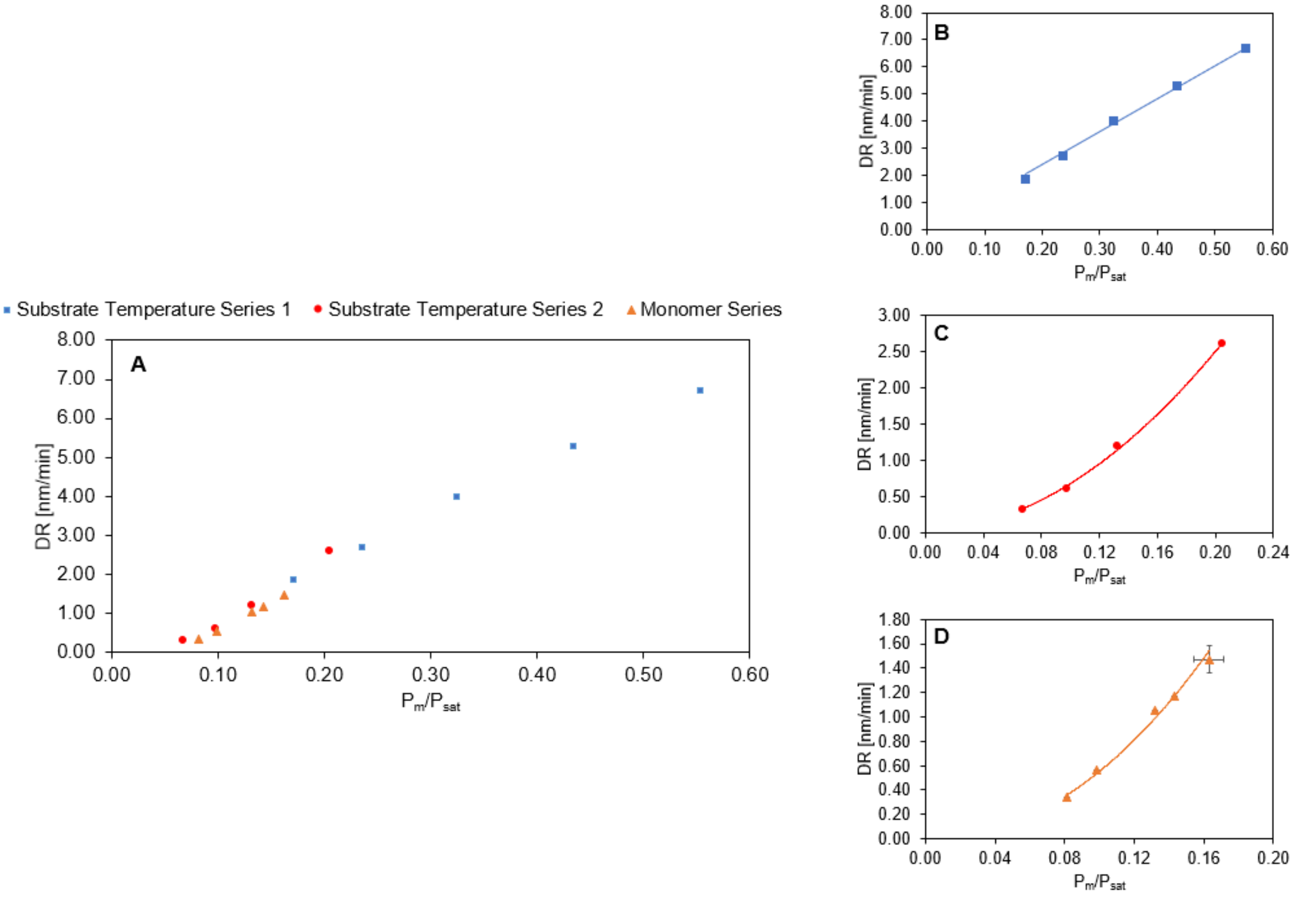

## 6 References


Asatekin, A., and Gleason, K. K. (2011). Polymeric nanopore membranes for hydrophobicity-based separations by conformal initiated chemical vapor deposition. *Nano Lett.* 11, 677–686. doi:10.1021/nl103799d.

Baxamusa, S. H., and Gleason, K. K. (2008). Thin polymer films with high step coverage in microtrenches by initiated CVD. *Chem. Vap. Depos.* 14, 313–318. doi:10.1002/cvde.200806713.

Lau, K. K. S., and Gleason, K. K. (2006a). Initiated Chemical Vapor Deposition (iCVD) of poly(alkyl acrylates): A kinetic model. *Macromolecules* 39, 3695–3703. doi:10.1021/ma0601621.

Lau, K. K. S., and Gleason, K. K. (2006b). Initiated Chemical Vapor Deposition (iCVD) of poly(alkyl acrylates): An experimental study. *Macromolecules* 39, 3688–3694. doi:10.1021/ma0601619.

Lebreton, P., and Boutevin, B. (2000). Primary radical termination and unimolecular termination in the heterogeneous polymerization of acrylamide initiated by a fluorinated azo-derivative initiator: A kinetic study. *J. Polym. Sci. Part A Polym. Chem.* 38, 1834–1843. doi:10.1002/(SICI)1099-0518(20000515)38:10<1834::AID-POLA690>3.0.CO;2-J.

Mao, Y., and Gleason, K. K. (2004). Hot Filament Chemical Vapor Deposition of Poly(glycidyl methacrylate) Thin Films Using tert-Butyl Peroxide as an Initiator. *Langmuir* 20, 2484–2488. doi:10.1021/la0359427.

Mitchell, M. J., Billingsley, M. M., Haley, R. M., Wechsler, M. E., Peppas, N. A., and Langer, R. (2020). Engineering precision nanoparticles for drug delivery. *Nat. Rev. Drug Discov.* doi:10.1038/s41573-020-0090-8.

Spencer, R. S., and Gilmore, G. D. (1949). Equation of state for polystyrene. *J. Appl. Phys.* 20, 502–506. doi:10.1063/1.1698417.

Xu, J., and Gleason, K. K. (2010). Conformal, amine-functionalized thin films by initiated chemical vapor deposition (iCVD) for hydrolytically stable microfluidic devices. *Chem. Mater.* doi:10.1021/cm903156a.
